# Sex differences in the association of body mass index with symptoms and cognitive deficits in Chinese patients with chronic schizophrenia

**DOI:** 10.1038/s41398-020-0717-x

**Published:** 2020-01-21

**Authors:** Chang Wei Wei, Ying Qi Chen, Mi Ma, Mei Hong Xiu, Xiang Yang Zhang

**Affiliations:** 1 Department of Anesthesiology, Beijing Chao-Yang Hospital, Capital Medical University, Beijing, P. R. China; 2 Qingdao Mental Health Center, Qingdao, P. R. China; 3 Peking University HuiLongGuan Clinical Medical School, Beijing HuiLongGuan Hospital, Beijing, P. R. China; 4 CAS Key Laboratory of Mental Health, Institute of Psychology, Chinese Academy of Sciences, Beijing, P. R. China; 5 Department of Psychology, University of Chinese Academy of Sciences, Beijing, P. R. China

**Keywords:** Psychiatric disorders, Pathogenesis

## Abstract

Accumulating studies have revealed gender differences in many aspects of schizophrenia (SZ), including obesity and cognitive function. The relationship between obesity and cognitive impairment in SZ has been studied before; however, the results are inconsistent. This study was designed to examine the sex differences in the relationship between body mass index (BMI) and cognitive deficits in Chinese patients with chronic SZ, which have not been investigated yet. 176 chronic patients with SZ (male/female = 108/68) and 200 controls (male/female = 120/80) were enrolled to compare the sex differences in cognitive functions measured by the Repeatable Battery for the Assessment of Neuropsychological Status (RBANS), BMI, and their associations. The clinical symptoms were evaluated using the positive and negative syndrome scales (PANSS). Our results showed that male patients had lower BMI and more negative symptoms but fewer positive symptoms than female patients (all *p* < 0.05). However, there was no significant sex difference in RBANS scores. In male patients, BMI was correlated with age of onset, PANSS general psychopathology, total score, negative symptom, together with RBANS language, visuospatial/construction, and attention. Further regression analysis showed that in male patients, BMI was significantly associated with RBANS language, PANSS general psychopathology, PANSS total score, and age of onset, with adjusted *R*^2^ = 0.22. These findings revealed significant sex differences in BMI, cognitive dysfunctions and their association in SZ. Nonetheless, these results should only be considered as preliminary because of the cross-sectional design, which will deserve further replication in first-episode patients using a prospective longitudinal design.

## Introduction

A growing body of studies have reported sex differences in almost all features of schizophrenia (SZ) from prevalence, incidence, prodromal symptoms, onset age, clinical manifestation, illness course, response to treatment, side effects, as well as long-term outcome, and social functions^1–4^. For example, the incidence of male SZ approximates 1.4 times of female patients^2^. Recent reviews reported that men had disadvantages over women in their earlier onset age and poorer response to antipsychotic treatments and poorer functioning^1,5,6^. Moreover, male patients displayed more clinical symptoms, especially negative symptoms^7^. The results suggest that men have a more severe form of SZ than women, which may be related to sex hormones^8^. However, several literatures reported no sex differences or even opposite results in some clinical presentations^9^.

Some researchers have specially examined sex differences in cognitive impairments of patients with SZ^1,10,11^. Previous studies showed that female patients showed better performance on executive functioning, verbal and language memory, and attention than male patients^10,12^. However, the results of sex differences in cognitive dysfunctions were inconsistent^9,13^, especially for those first-episode patients^3,4^.

SZ is related to a significantly high incidence of obesity^14–16^, and ~45–55% of patients with SZ were found to be obese^17–19^. One important cause for the increased rate of obesity among SZ patients is antipsychotic treatment, notably with olanzapine and clozapine^15,20–22^. Many studies have investigated sex differences in obesity as well as metabolic syndrome in SZ^14,23^. Some studies reported higher prevalence of obesity and metabolic symptoms in females than males^24–26^, with inconsistent results^27–29^.

Interestingly, recent studies have shown that obesity may produce negative effect on cognitive performance among healthy population^30,31^. Moreover, there is even increasing interest in examining the influence of obesity on cognitive deficits in SZ^32^. Only two recent studies explored their relationship. For example, one study reported that obesity was associated with poorer cognitive performance in Chinese SZ patients^28^. However, another study found no significant difference in cognitive dysfunction in SZ patients with and without obesity^33^. The real relationship between obesity and cognitive dysfunction in patients with SZ warrants further investigation.

In view of the high prevalence of obesity and cognitive impairments in SZ^34^, and significant sex differences in many aspects including obesity and cognitive deficits, it would be of great interest to examine whether there would be sex difference in the relationship of BMI and cognitive deficits in SZ, which has not been investigated in patients with SZ. Thus, this study was intended to explore whether there would be significant difference in the relationship of body mass index (BMI) with cognitive deficits in Chinese chronic SZ patients. We speculated that sex differences may occur in cognitive performance, BMI and their correlation in SZ.

## Methods

### Subjects

176 inpatients were randomly recruited from Beijing Hui-Long-Guan Hospital, a Beijing-city-owned psychiatric hospital. The hospital has 29 wards and the daily outpatient visits are about 500 patients. The inclusion criteria were: (1) aged 25–65 years, Han Chinese; (2) met the DSM-IV diagnosis of SZ, which was confirmed by 2 psychiatrists according to the Structured Clinical Interview for DSM-IV (SCID), without any patients with schizoaffective disorder; (3) at least 2 years of illness. The exclusion criteria were: (1) pregnancy or lactation; (2) major medical abnormalities; (3) alcohol or drug abuse or dependence; (4) subjects with ongoing infections, allergies or past history of autoimmune disorders; (5) subjects that took immunosuppressive drugs; (6) subjects with physical diseases or cerebral pathologies including multiple sclerosis seizure, dementia, epilepsy, Huntington’s disease, brain tumor, stroke, Parkinson’s disease, severe headache for unknown reasons, cardiovascular diseases; (7) education level less than 5 years by subject report. We had initially recruited 199 patients from Beijing Hui-Long-Guan Hospital. After screening, 5 individuals were excluded due to the inability to comprehend consent procedures, 10 patients were excluded for their severe medical abnormalities and 8 patients were excluded for being unable to perform cognitive tests. The excluded patients were not different from those included in the study in any demographic parameters. Thus, a total of 176 patients were randomly enrolled in the present study.

Research psychiatrists obtained social-demographic characteristics, smoking behavior and medical status using the questionnaires. Moreover, they collected a complete physical examination, laboratory tests and medical history for each subject. All patients had been receiving stable doses of oral antipsychotic drugs for at least six months before entry into the study. Antipsychotic drug treatment consisted mainly of drug monotherapy including clozapine (*n* = 80), risperidone (*n* = 38), chlorpromazine (*n* = 12), sulpiride (*n* = 9), perphenazine (*n* = 9), quetiapine (*n* = 7), haloperidol (*n* = 6), aripirazole (*n* = 5), and others (*n* = 10).

Since admission, all subjects received three balanced meals directly from the hospital canteen every day and took 60 min of physical exercise a day. The diet of all patients was similar. During hospitalization, their friends or family occasionally brought some snacks or fruits as a supplement.

Two hundred healthy controls (120 males and 80 females) were recruited by advertisements at the local community. A research psychiatrist ruled out a personal or family history of psychiatric disorder among healthy controls by direct psychiatric interview. Any subjects with medical illnesses or drug and alcohol abuse/dependence were excluded. The patients and healthy controls had a similar socioeconomic status and dietary patterns. Neither the patients nor the healthy controls had any history of alcohol or substance dependence (aside from tobacco). The protocol was approved by the Institutional Review Board (IRB). Psychiatrists carefully explained the protocol to those potential subjects. Each participant consented to join in the study.

### Clinical measurement and cognitive assessment

Four psychiatrists, who had simultaneously attended a training session in the use of the Positive and Negative Syndrome Scale (PANSS) before the study began, assessed the patient’s psychopathology using the PANSS. After training, repeated assessment showed that these psychiatrists maintained an interobserver correlation coefficient greater than 0.8 for the PANSS total score.

Cognitive functioning was evaluated using the Repeatable Battery for the Assessment of Neuropsychological Status (RBANS, Form A)^35^. The RBANS is comprised of 5 age-adjusted index scores and a total score. Test indices are immediate memory (comprising List Learning and Story Memory tasks); visuospatial/constructional (comprising Figure Copy and Line Orientation tasks); language (comprising Picture Naming and Semantic Fluency tasks); attention (comprising Digit Span and Coding tasks); and delayed memory (comprising List Recall, Story Recall, Figure Recall, and List Recognition tasks). Our group previously translated RBANS into Chinese and the clinical validity and its test-retest reliability were established among healthy controls and SZ patients^36^. The total and 5 index scores reported in this study were the standard scores.

### Assessment of anthropometric variables

Height and weight were measured and BMI was calculated. Participants in light indoor clothes were weighted by electronic scales. Height was measured to the closest millimeter and participants stood barefoot.

### Data analysis

Kolmogorov–Smirnov one-sample test was performed for normality. Continuous variables between groups were compared by analysis of variance and categorical variables by chi-square test (*X*^2^). To adjust the influence of age and education on cognitive function, analysis of covariance (ANCOVA) was further performed between groups. Then, we performed ANCOVA to compare the clinical characteristics between male and female patients. Associations of demographic, clinical characteristics, cognitive functions, and BMI were assessed by Pearson correlation coefficients in female and male patients separately. Logistic regression analyses were performed to explore which characteristics were related to BMI. Adjusting for multiple testing, the Bonferroni corrections were used. Statistical analysis was performed with SPSS (version 20.0) and significance levels of *p*-values were set at 0.05.

## Results

### Sample characteristics in patients and controls grouped by sex

Table 1 shows that smoking was more common in male patients and male controls than female counterparts. Further, male patients smoked more than male controls (77.1% vs 59.2%); however, female patients smoked fewer than female controls (7.4% vs 25.0%) (all *p* < 0.01). Therefore, smoking was controlled in the following analyses.

**Table 1:** Demographics and cognitive function in SZ and healthy controls.

	SZ		Healthy control				
	Male (*n* = 108)	Female (*n* = 68)	Male (*n* = 120)	Female (*n* = 80)	Diagnose F (*p*-value)^a^	Sex F (*p*-value)^a^	Diagnose × Sex F (*p*-value)
Age (years)	51.5 ± 9.1	48.3 ± 11.7	49.9 ± 9.6	48.7 ± 10.0	0.30 (0.58)	4.62 (0.03)	0.93 (0.34)
Education (years)	9.6 ± 2.5	10.4 ± 2.6	9.5 ± 3.0	9.9 ± 2.7	1.14 (0.29)	4.73 (0.03)	0.59 (0.44)
Body mass index (BMI)	24.2 ± 4.7	25.6 ± 4.3	25.7 ± 3.3	24.2 ± 3.1	0.03 (0.87)	0.26 (0.61)	8.49 (0.004)
Smoker (*n*%)	83/76.9%	5/7.4%	71/59.2%	20/25.0%	0.96 (0.45)	93.0 (0.000)	90.4 (0.000)
RBANS							
Immediate memory	62.9 ± 17.9	66.3 ± 19.8	72.8 ± 16.8	75.7 ± 17.7	17.46 (<0.0001)	1.88 (0.17)	0.02 (0.90)
Visuospatial/constructional	82.6 ± 18.3	84.8 ± 20.1	81.0 ± 15.0	80.8 ± 14.8	1.74 (0.19)	0.24 (0.63)	0.32 (0.57)
Language	85.7 ± 13.0	86.7 ± 14.6	95.3 ± 10.5	94.8 ± 12.2	31.3 (<0.001)	0.03 (0.87)	0.20 (0.66)
Attention	81.9 ± 15.0	82.3 ± 15.0	86.7 ± 17.0	84.4 ± 19.7	2.42 (0.12)	0.19 (0.66)	0.34 (0.56)
Delayed memory	69.2 ± 19.6	73.5 ± 22.1	85.7 ± 15.5	87.3 ± 14.0	46.0 (<0.001)	1.78 (0.18)	0.37 (0.55)
Total score	69.6 ± 16.5	73.5 ± 17.8	79.5 ± 13.2	79.7 ± 15.0	16.7 (<0.001)	1.16 (0.28)	0.90 (0.34)

^a^The *p*-values for RBANS and BMI were adjusted for age and education as covariates.

There was a significant diagnosis × sex interaction on BMI. Further analysis showed that male patients had lower BMI than females (F = 4.19, df = 1, 175, *p* = 0.042), while male controls had significantly higher BMI than female controls (F = 8.87, df = 1, 198, *p* = 0.002).

As shown in Table 2, male patients had older age, younger age of onset, poorer education, together with more negative symptoms but less positive symptoms compared to female patients (all *p* < 0.05).

**Table 2:** Characteristics of male and female patients with SZ.

	Male	Female	F or *X*^2^	*p*-value
Age	51.5 ± 9.1	48.3 ± 11.7	4.4	0.04
Education (years)	9.6 ± 2.5	10.4 ± 2.6	4.6	0.032
Age of onset (years)	23.9 ± 5.5	26.0 ± 7.2	4.75	0.031
Antipsychotic types (typicals/atypicals)	24/83	13/56	0.33	0.57
Antipsychotic dose (CPZ equivalents)	411.2 ± 193.8	437.6 ± 251.9	−0.76	0.45
Duration of current antipsychotic treatment	66.6 ± 68.5	46.3 ± 68.3	1.75	0.08
Duration of current antipsychotic treatment	66.6 ± 68.5	46.3 ± 68.3	1.75	0.08
Number of hospitalizations	3.9 ± 3.7	3.5 ± 2.4	0.73	0.46
PANSS				
Positive symptom scale	12.0 ± 4.7	14.6 ± 6.2	−3.20	0.002
Negative symptom scale	23.0 ± 6.8	20.4 ± 9.0	2.05	0.04
General psychopathology scale	27.3 ± 4.9	27.9 ± 6.0	−0.66	0.51
Total score	62.3 ± 12.3	62.9 ± 15.3	−0.50	0.62

Mean ± SD.

*CPZ* chlorpromazine, *PANSS* the Positive and Negative Syndrome Scale.

### Cognitive function performance in SZ and controls grouped by sex

Although female patients appeared to show higher scores on RBANS total score, delayed memory and immediate memory scores, as compared to male patients, these differences were not statistically significant (all *p* > 0.05). Also, there was no sex difference in cognitive function performance in controls (all *p* > 0.05) (Table 1).

### Sex differences in the associations between BMI and clinical symptoms and cognitive measures

For all patients, BMI was correlated with age of onset (*r* = 0.15, df = 177, *p* = 0.041), Language (*r* = 0.26, df = 111, df = 0.006, df = 111), Visuospatial/construction (*r* = 0.19, df = 111, *p* = 0.04), negative symptom (*r* = −0.20, df = 173, *p* = 0.009), general psychopathology (*r* = −0.23, df = 173, *p* = 0.002), and PANSS total score (*r* = −0.24, df = 173, *p* = 0.002).

**Fig. 1:**
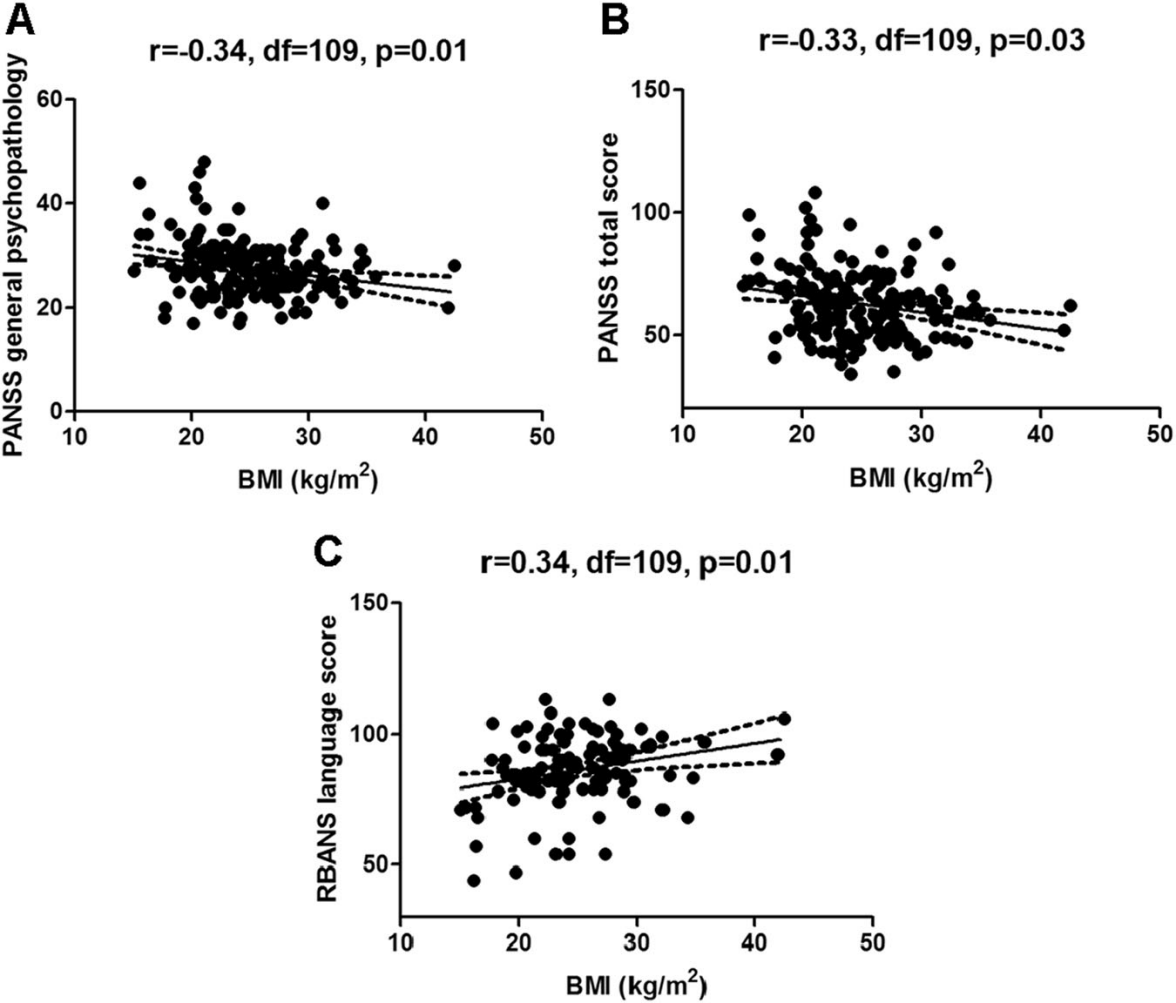
There were significant negative relationships between body mass index (BMI) and PANSS general psychopathology (*r* = −0.34, df = 108, *p* = 0.01, (**a**) and total score (*r* = −0.33, df = 108, *p* = 0.03, (**b**), as well as a positive relationship between BMI and RBANS language score (*r* = 0.34, df = 108, *p* = 0.01, **(c**) in male schizophrenia patients.

As shown in Table 3, BMI was related to the following characteristics: onset age (*r* = 0.21, df = 109, *p* = 0.03), PANSS general psychopathology (*r* = −0.34, df = 109, *p* < 0.001), total score (*r* = −0.33, df = 109, *p* < 0.001), negative symptom (*r* = −0.23, df = 109, *p* = 0.02), together with RBANS language (*r* = 0.34, df = 109, *p* < 0.001), visuospatial/construction (*r* = 0.27, df = 109, *p* = 0.01) and attention (*r* = 0.25, df = 109, *p* = 0.02) in male SZ subjects. Further stepwise regression analysis indicated that BMI was significantly associated with RBANS language (beta = 0.11, *t* = 2.65, *p* = 0.01), PANSS general psychopathology (beta = −0.27, *t* = −2.64, *p* = 0.01) and PANSS total score (beta = −0.09, *t* = −2.21, *p* = 0.03), with adjusted *R*^2^ = 0.22 (Fig. 1).

**Table 3:** Correlations between BMI and clinical variables or cognitive performance measures in male and female patients with SZ^a^.

	Male (*n* = 108)	Female (*n* = 68)
Age (years)	−0.08 (0.42)	0.10 (0.41)
Education (years)	−0.01 (0.93)	−0.01 (0.93)
Age of onset (years)	0.21 (0.03)	0.08 (0.50)
PANSS		
Positive	−0.14 (0.16)	−0.04 (0.75)
Negative	−0.23 (0.02)	−0.12 (0.35)
General psychopathology	−0.34 (<0.001)	−0.10 (0.41)
Total	−0.32 (<0.01)	−0.15 (0.23)
RBANS		
Immediate memory	0.11 (0.30)	−0.18 (0.36)
Visuospatial/constructional	0.27 (0.01)	−0.07 (0.71)
Language	0.34 (0.001)	−0.01 (0.95)
Attention	0.25 (0.02)	−0.16 (0.41)
Delayed memory	0.13 (0.25)	−0.11 (0.59)
Total score	0.19 (0.09)	−0.13 (0.50)

^a^Values are shown as r (p).

Nonetheless, no statistically significant correlation between BMI and clinical and cognitive parameters was found in the female patients (all *p* > 0.05). Furthermore, no correlation between BMI and cognitive domains was found across the entire healthy subjects or when analyzed by sex group (all *p* > 0.05).

## Discussion

In our present study, significant sex differences were found in many aspects of SZ, showing that male patients had older age, younger age of onset, poorer education, together with more negative symptoms but less positive symptoms compared to female patients. Although female patients appeared to show high scores on RBANS immediate memory, delayed memory and total scores compared with males, there was no statistically significant sex difference in the RBANS scores. Notably, we did find negative associations of BMI with negative symptom, general psychopathology, and PANSS total score, as well as positive associations between BMI and age of illness onset, RBANS language, visuospatial/construction, and attention in SZ patients. More importantly, we found that these significant associations were largely driven by the male patients, suggesting that the influence of BMI on cognitive performance and clinical symptoms may be mainly present in male patients with SZ.

In this present study, we found that female patients had higher education level and later age of onset than male patients, which was consistent with previous reports showing that women with SZ received better education^3,37,38^. However, some reports did not find sex difference in onset age of SZ^39,40^. Also, we found that male patients had severer negative symptoms and less positive symptoms than female patients, which confirmed other studies^3,39,41–43^. However, there are some conflicting results in the investigation of sex differences in clinical manifestations of SZ^9^. These discrepant results may be due to different stages of disease (first episode vs. chronic), sample representation (community vs. outpatients vs. inpatients), exposure for antipsychotics, or the different ethnic population^3^.

Interestingly, we found that female patients exhibited higher BMI than male patients, which is accordant with previous studies^26,44^. However, a recent study reported the contrast result in a Chinese population^28^. We scrutinized their methods and found that the subjects in their study were much younger compared to the subjects in our current study (around 30 years vs. 50 years). Moreover, their subjects had much shorter duration of illness than ours (about 2.2 years vs 25 years). Thus, our patients had taken antipsychotic medicines much longer and may have had more side effects related to the change of sex hormones caused by long-term antipsychotic treatment. Moreover, recent studies reported that the hormonal changes during menopause increased abdominal obesity, insulin resistance and hyperplasia markedly^45–47^. Also, a previous study showed that menopausal women developed much more obesity and other metabolic abnormalities than non-menopausal women^48^. Our female patients had an average age of 48.3 years and most of them were either postmenopausal or perimenopausal. Taken together, our finding of higher BMI in female patients may be related with older age and possible hormone alterations due to long-term antipsychotic treatment as well as postmenopause or perimenopause. However, this is only our speculation. The relationship between gender and BMI deserves further investigation in large-scale follow-up studies.

Furthermore, we found significantly inverse associations between BMI and clinical symptoms, accordant to some prior studies^49–51^. This association may be caused by treatments with antipsychotics, since the antipsychotic treatments may improve the psychopathological symptoms of SZ patients and simultaneously cause the increase in weight due to their side effects^49^. Previous studies found that antipsychotic treatment, such as clozapine, olanzapine, and haloperidol decreased the psychological symptoms accompanying weight gain^49,52–56^. In particular, our current study revealed that BMI was positively related to onset age of SZ, suggesting a protective factor for the onset of SZ. Taken together, these findings suggest that high BMI or increased bodyweight during antipsychotic treatment may be beneficial to the clinical symptoms of SZ patients.

Another interesting finding was that BMI showed significantly positive association with cognitive performance on Language and visuospatial/construction domains. Gunstad et al. demonstrated that elevated BMI was associated with better performance on psychomotor speed, visuospatial skills, and attention^57^, which is similar to our finding. Moreover, higher BMI was found to be associated with better cognition in older adults among the general population, but low BMI or weight loss preceded dementia^58,59^. However, our finding is in disagreement with two recent studies reporting no association of high BMI or obesity with cognitive performance^33^ or even an inverse association between higher BMI and lower scores on some cognitive performance in SZ patients^28^. Such a discrepancy of the relationship between BMI and cognitive functioning due to the complicated inter-relationships between BMI/obesity and cognition warrants further exploration in future studies.

More importantly, we found significant associations between BMI and age of onset, clinical symptoms and cognitive function in SZ only in men, but not in women. Further scrutiny on all *p*-values for associations of BMI with clinical and cognitive parameters in whole patients and in males, it indicated that almost all *p*-values were increased in males compared to those in all patients, further suggesting that high BMI may produce stronger impacts on clinical and cognitive parameters only in male patients. The finding of a relationship between sex and cognitive function in SZ patients may be explained by sex hormones. Prior researches showed significant correlations of sex hormones with anthropometric parameters^60,61^. For example, a recent literature demonstrated that serum levels of testosterone were inversely related to all anthropometric measures including leptin, waist-to-hip-ratio, and BMI in men, while in women, testosterone was positively correlated with BMI^61^. On the other hand, basic and clinical studies demonstrated that hypoandrogenism was associated with cognitive impairment. For example, some animal studies found a relationship between the decrease of androgens including dehydroepiandrosterone (DHEA) and its sulfate (DHEA-S) as well as testosterone and memory impairment during aging^62,63^. Therefore, some animal studies showed that the administration of these hormones improved the performance of cognitive tasks^64^. In the brain, these androgens promote neuroprotection, neurite growth, neurogenesis, neuronal survival, and catecholamine synthesis and release, and they modulate physiological functions, such as sexual behavior, diet, emotion, and cognition^64,65^. Furthermore, testosterone was found to be positive with the execution of spatial, semantic, working, and verbal episodic memory in elderly subjects^66,67^. Taken together, androgen was closely correlated with both BMI and cognitive performance. Therefore, the finding of a positive relationship between BMI and some cognitive domains including language, visuospatial/construction and attention only in male patients may be associated with high levels of androgen hormone. However, it is worth mentioning that no sex difference was observed in the association between BMI and cognitive function in controls. Currently, it is unclear why sex difference in association between BMI and cognition was only observed in SZ patients, while not in controls. In addition, we could not explain the findings that BMI was only associated with some cognitive domains, but not with others such as immediate or delayed memories.

We have several limitations in this present study. First, it is only a cross-sectional study, which does not allow us to make causal inferences on the relationship between BMI and cognition in men and women with SZ. Second, the sample size is relatively small, which may explain the negative result in the relationships between BMI and cognition in female patients. Moreover, the ratio of males vs. females was unbalanced in patient group, which may lead to the statistical basis. Third, the age of the patients ranged from 25–65 years, and the effect of the age heterogeneity on cognitive performance is obvious. Fourth, all patients were of chronic types and on long-term different medications. Since the antipsychotic drugs such as atypical antipsychotics have more metabolic side effects and different effects on cognitive function in SZ patients compared to typical antipsychotics, we could not rule out totally the differential influences of different antipsychotic medications on BMI and cognition, as well as on their association, although we controlled for antipsychotic treatment as a confounding factor in the statistical analyses. Fifth, in this study, we utilized RBANS for cognition, rather than the Brief Assessment of Cognition in Schizophrenia (BACS) or Measurement and Treatment Research to Improve Cognition in Schizophrenia (MATRICS) that represent the gold standard for cognition in schizophrenia. This is because when we carried out this study, we did not have BACS or MATRICS at that time. Sixth, only one hospital in China was included in this survey. Therefore, the findings could not be generalized to other settings.

In summary, sex difference was found in many aspects of SZ patients in our current study. Moreover, we observed significantly negative associations between BMI and clinical symptoms including PANSS general psychopathology, negative symptom, and total score, but positive associations between BMI and age of illness onset, or some cognitive performance including RBANS language, visuospatial/construction and attention. More importantly, these significant associations were largely driven by the male patients. These sexually differential associations between BMI and cognition may be related to sex hormones particularly testosterone, since the androgens were found to be associated with both anthropometric measures and with cognitive functioning. However, we did not find these sexually differential associations in healthy controls.

Considering the limitations of the current study, our results should be preliminary and should be verified using a longitudinal study of the first-episode patients with SZ.
